# Inclusion of periodontal ligament fibres in mandibular finite element models leads to an increase in alveolar bone strains

**DOI:** 10.1371/journal.pone.0188707

**Published:** 2017-11-30

**Authors:** Steven W. McCormack, Ulrich Witzel, Peter J. Watson, Michael J. Fagan, Flora Gröning

**Affiliations:** 1 Medical and Biological Engineering Research Group, School of Engineering and Computer Science, University of Hull, Hull, United Kingdom; 2 Fakultät für Maschinenbau, Ruhr-Universität Bochum, Universitätsstraße 150, Bochum, Germany; 3 Arthritis and Musculoskeletal Medicine Research Programme, School of Medicine, Medical Sciences and Nutrition, University of Aberdeen, Aberdeen, United Kingdom; The University of Adelaide, AUSTRALIA

## Abstract

Alveolar bone remodelling is vital for the success of dental implants and orthodontic treatments. However, the underlying biomechanical mechanisms, in particular the function of the periodontal ligament (PDL) in bone loading and remodelling, are not well understood. The PDL is a soft fibrous connective tissue that joins the tooth root to the alveolar bone and plays a critical role in the transmission of loads from the tooth to the surrounding bone. However, due to its complex structure, small size and location within the tooth socket it is difficult to study *in vivo*. Finite element analysis (FEA) is an ideal tool with which to investigate the role of the PDL, however inclusion of the PDL in FE models is complex and time consuming, therefore consideration must be given to how it is included. The aim of this study was to investigate the effects of including the PDL and its fibrous structure in mandibular finite element models. A high-resolution model of a human molar region was created from micro-computed tomography scans. This is the first time that the fibrous structure of the PDL has been included in a model with realistic tooth and bone geometry. The results show that omission of the PDL creates a more rigid model, reducing the strains observed in the mandibular corpus which are of interest when considering mandibular functional morphology. How the PDL is modelled also affects the strains. The inclusion of PDL fibres alters the strains in the mandibular bone, increasing the strains in the tooth socket compared to PDL modelled without fibres. As strains in the alveolar bone are thought to play a key role in bone remodelling during orthodontic tooth movement, future FE analyses aimed at improving our understanding and management of orthodontic treatment should include the fibrous structure of the PDL.

## 1. Introduction

One of the most widely used methods for theoretical analysis of biological structures is finite element analysis (FEA) [1]. FEA is a “full-field technique”, capable of showing stresses and strains throughout the whole structure, and thus is a useful tool for studying the relationship between the forces applied to a bone and its morphology in a non-destructive way. However, producing accurate representations of biological structures in FE models is very challenging. The validity of such models is highly dependent on faithful representations of the geometry and material properties of the structure, as well as the application of the correct loading and boundary conditions [2]. Biomechanical FE models are often produced from micro-computed tomography (μCT) data and, although image processing can introduce some errors, this should lead to models which are accurate geometric representations of the biological structures [3]. However, determining realistic material properties, loading and boundary conditions remains a major challenge for biomechanical modelling.

In any FE model of the mandible (i.e. the whole mandible or a section of the mandible), there are several different materials which need to be modelled. Depending on the size and level of complexity of the model, materials which are commonly considered include enamel, dentine, periodontal ligament (PDL), cortical bone and trabecular bone. Although there are many components involved, the PDL causes arguably the most difficulty in dental FE models [4]. The PDL is a soft fibrous connective tissue which joins the cementum of the tooth root to the alveolar bone thus anchoring the tooth in its socket [5, 6]. The primary function of the PDL is to secure the tooth in its socket [7]. Due to its location, the PDL plays an important role in the transmission of masticatory and orthodontic loads from the teeth to the surrounding bone [8, 9]. It is also important for tooth mobility since it has a low stiffness compared to the materials around it [10].

The PDL contains both elastic components, mainly collagen fibres, and fluid components such as blood and lymph vessels and interstitial fluid [11]. MicroCT scans of the mandible in this study and previous studies, for example [10, 12], shows that the thickness of the PDL varies around the tooth root, but it is typically 0.25mm thick, with the fibres making up around 50 to 75% of the volume of the tissue [5, 6]. The collagen fibres are grouped together in principal fibre bundles and form a meshwork like a stretched fishing net extending between the cementum and alveolar bone [13–15]. The complex arrangement of fibres ensures that regardless of the direction of force applied, some fibre bundles are always placed in tension [14]. The fibres are also thought to transmit vertical forces from the teeth as lateral forces to the tooth socket and in doing so, help to prevent high stresses occurring at the apex of the tooth root [16]. The PDL has been shown to have nonlinear, viscoelastic material properties which vary at different locations and in different directions along the tooth root [11, 14, 17]. However, whilst attempts have been made to characterise the material properties of the whole PDL, little is known about the specific material properties or geometry of the individual PDL fibres [8].

When simulating masticatory loading of the mandible in a FE model, the way in which the PDL is modelled can have a considerable influence on the results produced. Whether to include the PDL and what material properties to assign if it is included, are the subject of much debate throughout the literature [18–20]. When it is included, most models idealise the PDL as a layer of solid, homogeneous and isotropic material [21]. Although some authors have attempted to represent its material properties more accurately, for example [17, 22, 23], only a few have attempted to include its fibrous structure [8, 16, 24–28]. So far, those models which have included the PDL fibres only investigated their effect under low loads, typical of orthodontic tooth movement, and in models with only a single tooth.

Many experimental studies have shown that bone mass and structure is adapted to its mechanical environment and that strain magnitudes (among other factors such as strain frequency) play a key role in this adaptation. Bone formation has been linked to an increase in strain magnitude, *e*.*g*. from a new exercise regime, whereas bone resorption is often associated with a decrease in strain magnitudes, *e*.*g*. due to prolonged bed rest [29–35]. One important clinical consequence of mechanical adaptation of bone is orthodontic tooth movement, which occurs due to site-specific resorption and formation of alveolar bone [17]. The crown loading conditions required to move teeth during orthodontic treatment are well understood [36], typically with low continuous forces of around one Newton applied for weeks at a time [7]. Load transfer from the teeth to the surrounding bone is clearly influenced by the PDL. Whilst bone remodelling has been widely investigated in long bones, the presence of the PDL makes direct application of these theories to alveolar bone remodelling more difficult.

Whether orthodontic tooth movement is triggered by strains in the alveolar bone or in the PDL remains the subject of much debate within the literature [22]. In the case of PDL controlled orthodontic tooth movement, it is suggested that tooth displacement alters the vascularity and blood flow within the PDL, which initiates biochemical and cellular activities that cause local bone adaptation [37]. For alveolar bone controlled orthodontic tooth movement, it is suggested that alveolar bone adaptation is caused by strains in the alveolar bone through the same mechanisms that cause functional bone adaptation in other bones [37].

Different hypotheses have been suggested regarding the biomechanical nature of orthodontic tooth movement. According to the “pressure-tension hypothesis” [38] tooth movement in the direction of the applied load compresses the PDL on the side to which the tooth is moved and stretches it on the opposite side. This leads to symmetric zones of compression and tension occurring in the periodontium, with the compression leading to bone resorption and tension causing bone formation [17, 22]. A second hypothesis regarding orthodontic tooth movement is the “alveolar bending hypothesis” first reported by Baumrind [39]. It suggests that as well as deforming the PDL, tooth movement also causes deformation of the alveolar bone. The walls of the tooth socket are thought to behave like cantilever beams with bone being added to the compressive surfaces and removed from the tensile surfaces. More recently, a third hypothesis, the “stretched fibre hypothesis” [40] has been suggested to match orthodontic tooth movement with orthopaedic bone remodelling in accordance with Frost’s mechanostat theory [41]. Melsen [40] suggests that the PDL fibres will be compressed on the side to which the tooth is pushed causing an area of low strain and bone resorption, and stretched on the opposite side causing high strain and bone formation. If this hypothesis is correct, then it would be important to include the fibres of the PDL in FE models, especially when investigating orthodontic tooth movement (see McCormack *et al*. [42] for a more detailed description and diagram of these hypotheses).

Previous FE models have shown strains in the alveolar bone to be much lower than those predicted by Frost’s mechanostat, for example [36, 43–45]. Due to this, it has been suggested that orthodontic tooth movement is triggered by strains in the PDL rather than strains in the alveolar bone [46]. However, synchrotron studies by Dalstra *et al*. [47, 48] showed that the surface of the alveolar bone is not smooth and so they suggested that FE models might have underestimated the strains in the tooth sockets by not accounting for local stress and strain concentrations due to the structure of the alveolar bone. Therefore, more detailed FE models are required to better understand the strains caused by orthodontic tooth movement.

The aim of the current study was to investigate the effect of including the PDL in mandibular FE models under occlusal and orthodontic loads, and the effect of including the fibrous structure of the PDL in these models. The purpose is to increase our understanding of the stresses and strains in the bone under these loads (which are currently impossible to measure directly *in vivo* in humans) and consider how the bone might adapts to them. The results of our previous study [42] indicated that the way in which the PDL is modelled affects strains in the tooth socket. However, since we used a simplified single tooth model in that previous study, we did not consider the geometry of the alveolar bone in detail (e.g. the trabecular bone architecture) and the results could not reveal how the inclusion of the PDL affects strains elsewhere in the mandible. This study builds upon the approach by McCormack *et al*. [42], applying a similar technique for representing the fibrous structure of the PDL, but using a FE model with realistic geometry based on a μCT scan of the molar region of a human mandible. Three different methods of modelling the PDL were compared (fibrous, solid, and no PDL), and strains were examined both adjacent to and further from the tooth socket of the loaded tooth.

## 2. Materials and methods

### 2.1 Finite element model creation

A μCT scan of a dry adult human right hemi-mandible specimen was obtained using an X-Tek HMX160 μCT scanner (X-Tek Systems Ltd., Tring, UK). (The use of the specimen was approved by the HTA Designated Individual at the Hull York Medical School according to the Human Tissue Act 2004). The μCT data set was exported as a stack of 8-bit TIFF (tagged image file format) images with a voxel size of 0.040 mm in all three directions, and imported into AVIZO (version 6.3, FEI Visualization Sciences Group, Berlin, Germany) for image segmentation.

Since a detailed representation of the three-dimensional geometry of the trabeculae was to be included in the FE models, a Ray Casting Algorithm (RCA) method [49] was used for automatic thresholding of the grey scale values. This method was developed by Scherf and Tilgner [49] specifically for segmenting fine scale bone structures, and they found it to be superior to traditional methods such the half-maximum height thresholding protocol [50–52] or the adaptive iterative thresholding method [53–55].

**Fig 1:**
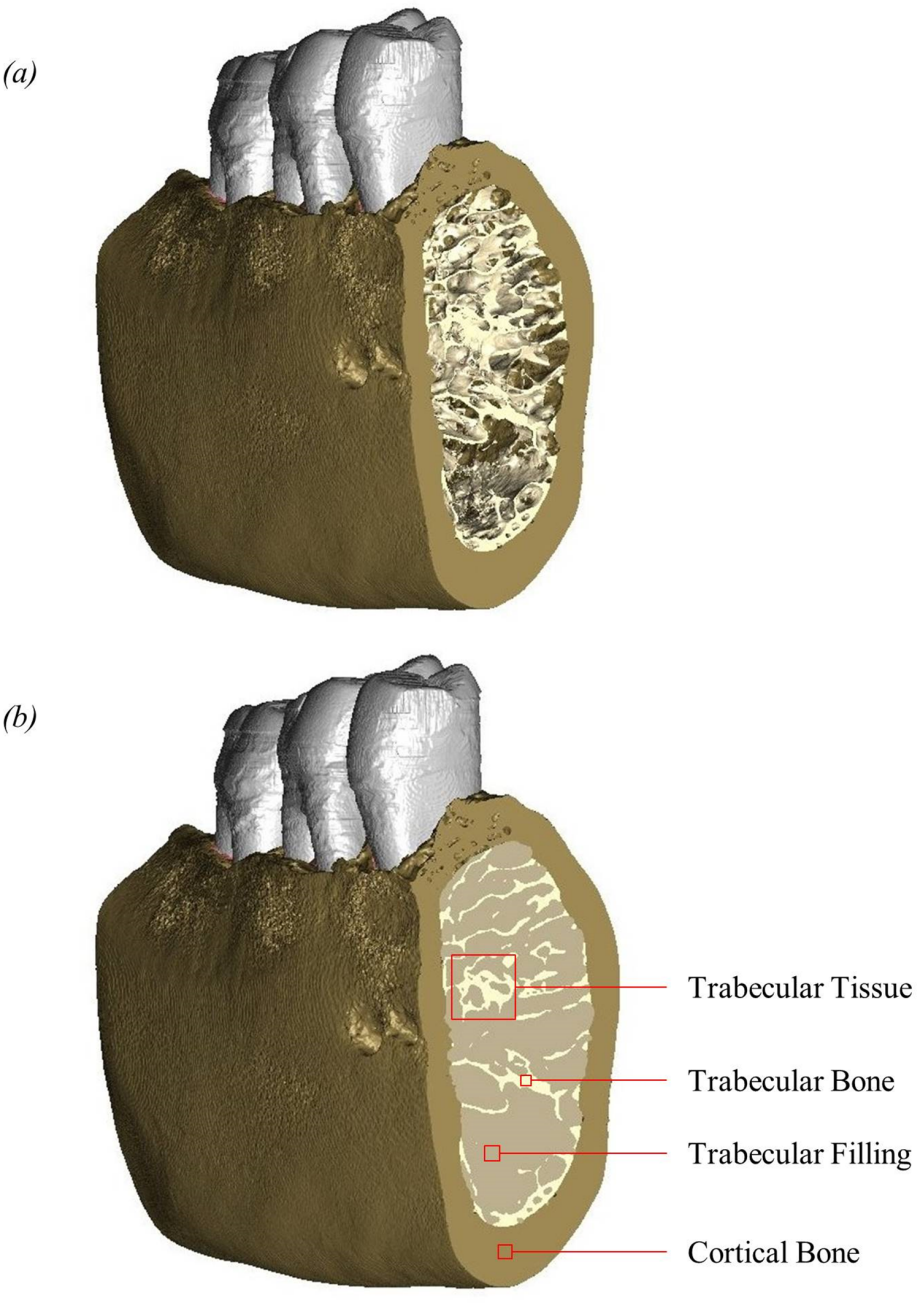
Surface models showing the buccal side and the cut mesial surface of the mandibular bone. (a) cortical bone and trabecular bone without trabecular filling; (b) cortical bone and trabecular bone with trabecular filling. Trabecular bone refers to the material forming the individual trabeculae. Trabecular filling refers to the material in the pores surrounding the trabeculae. Trabecular tissue refers to the combination of trabecular bone and trabecular filling. Cortical bone refers to the dense bone around the outer surface of the mandible.

The model contained just the molar region including the second premolar and first and second molars. Pre-mortem loss of the third molar meant it was not present in this specimen, and could, therefore, not be included in these models. Further manual segmentation was required to separate different materials within the model. A material was added to fill the space between each individual tooth and alveolar bone, to represent the PDL. This meant the PDL material completely surrounded the tooth root and joined the tooth root to the alveolar bone for all three teeth. Cortical and trabecular bone regions were also separated from each other, with alveolar bone being defined the same as cortical bone. A material was then added to fill the space between the trabeculae, creating a combined structure to represent the trabecular tissue as shown in Fig 1. A tetrahedral mesh was then generated and exported to ANSYS (version 14.5, ANSYS Inc., Canonsburg, PA, USA) for FEA.

Solid 10-noded high-order tetrahedral elements (SOLID187) [56] were used throughout the model. In total, the model included 1,338,912 solid elements and contained five different materials: cortical bone, trabecular bone, trabecular filling, teeth and PDL. Additional elements representing PDL fibres were added later. The different materials of the teeth were not modelled.

For this study we use cortical bone to refer to the dense bone around the outer surface of the mandible; ‘trabecular bone’ refers to the material forming the individual trabeculae; ‘trabecular filling’ refers to the material in the pores surrounding the individual trabeculae; and ‘trabecular tissue’ refers to the trabecular bone and trabecular filling combined. These terms are illustrated in Fig 1. Using these materials, we could model the trabecular tissue in two different ways, by adjusting the material properties assigned to the trabecular bone and the trabecular filling within the model: as trabecular structure (i.e. with the individual trabeculae modelled) and as bulk trabecular material (i.e. trabecular bone and trabecular filling combined). As well as the two different types of trabecular tissue, three different types of PDL were investigated: fibrous PDL, solid PDL, and no PDL. Therefore, in total six different types of models were created: (1) Fibrous PDL and trabecular structure; (2) Fibrous PDL and bulk trabecular material; (3) Solid PDL and trabecular structure; (4) Solid PDL and bulk trabecular material; (5) No PDL and trabecular structure; (6) No PDL and bulk trabecular material. Thus all six models were created from the same FE mesh by varying the material properties assigned to different elements, with additional link elements included in the fibrous PDL models.

**Fig 2:**
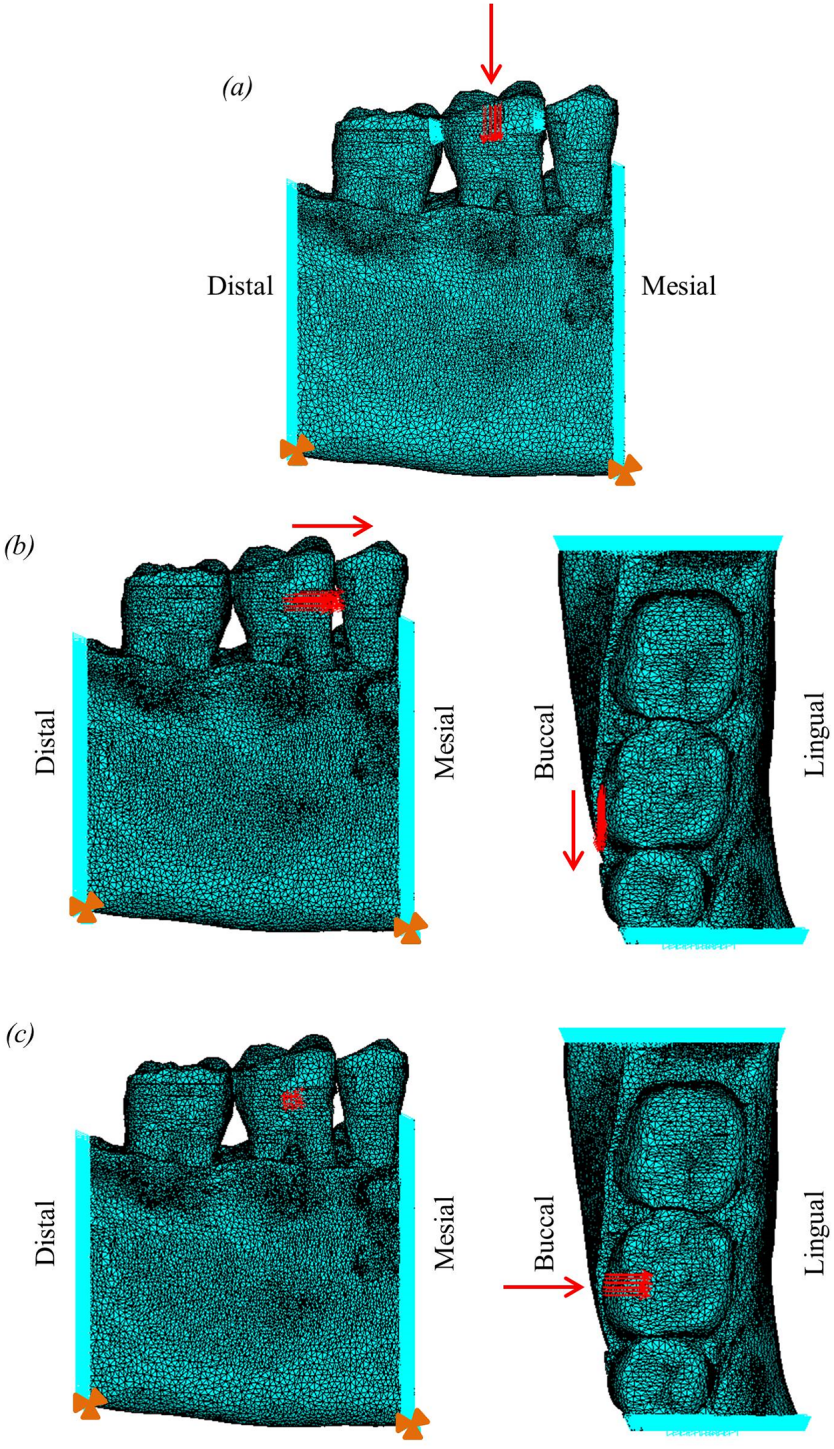
Finite element model, showing loading and boundary conditions applied for occlusal and orthodontic loads, where light blue triangles represent constraints and red arrows represent applied forces. (a) occlusal load; (b) two views of the distomesial orthodontic load; (c) two views of the buccolingual orthodontic load. Additional red arrows highlight the direction of the applied load, and orange triangles indicate the nodes at the base constrained in all degrees of freedom.

The boundary conditions applied to the model are shown in Fig 2. All nodes on the two cut surfaces at the mesial and distal ends of the model were constrained in the mesiodistal direction to represent the adjacent bone in this direction in a whole mandible. A small number of nodes at the base of each edge were constrained in all three directions to prevent rigid body translation of the model in the dorsoventral direction. In addition, for the occlusal load, a selection of nodes on each of the mesial and distal sides of the exterior surface of the tooth crown on the middle tooth were constrained in the mesiodistal direction to represent the points of contact between adjacent teeth, which would limit movement in that direction, but allow dorsoventral displacement. The constraints on the side of the molar tooth were removed for the two orthodontic loads since rotation of the tooth may occur during orthodontic tooth movement.

Three separate loads were applied to the models: an occlusal load and two different orthodontic loads. For the occlusal load, a total load of 500N was distributed over a small area around the centre of the occlusal surface of the crown of the first molar, directed in the corono-apical direction (Fig 2a). A load of 500N was chosen to represent a maximal human bite force [57–59]. A maximum bite force was chosen, rather than a typical (daily) bite force, to allow confirmation that the performance of the system was reasonable up to the peak force that it might experience. While this leads to strains that are higher than those experienced during every day loading, this isn’t important since the main purpose of the study is to examine relative differences between models rather than to examine absolute strain values. Two separate orthodontic loads were applied, one directed in the mesiodistal direction, and one in the buccolingual direction. A 1N load was distributed over a small area around the centre of the buccal surface of the crown of the first molar (Fig 2b and 2c). The value of 1N was chosen to represent a typical load used during orthodontic treatment [60].

Previous FEA studies of whole human mandibles using tetrahedral finite element meshes have included convergence tests, for example [61–63], and typically found that convergence was reached with around 100,000 elements, which is much less than that used in this model of just the molar region. We checked for convergence by comparing the results of this model, with around 1 million elements, to results from a model with around 4 million elements. We compared maximum principal strain, minimum principal strain and Von Mises strain at five locations on the outer surface of the cortical bone and found that the results only varied by up to 5% at the same locations in the two models [results not included here].

**Fig 3:**
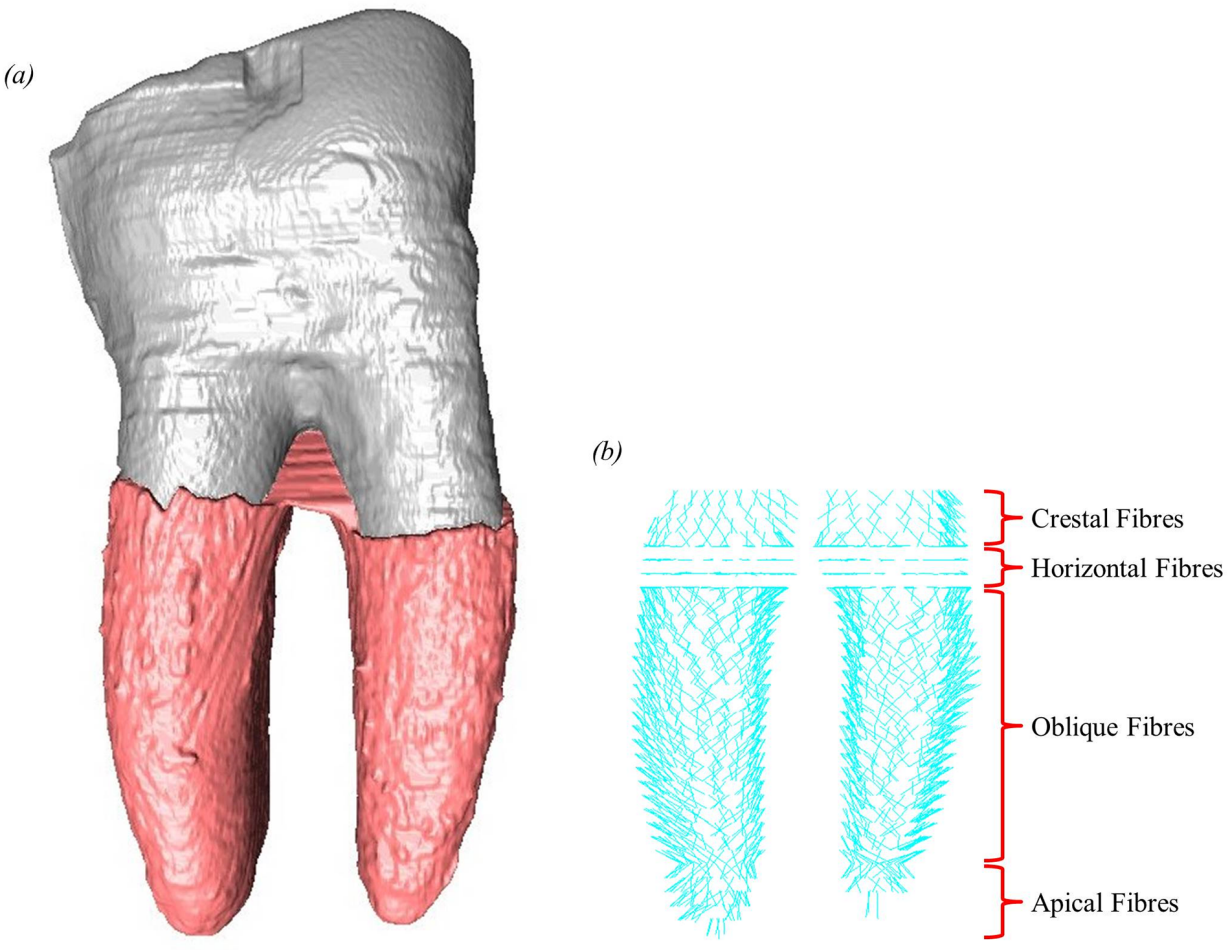
Buccal side view of 2,112 link elements, added to the fibrous PDL models, connecting the alveolar bone to the tooth root through the PDL. (a) surface model showing first molar and surrounding PDL; (b) link elements representing PDL fibres.

The six different models were created by varying the material properties assigned to different elements, with or without the addition of PDL fibres. All materials in the models were assigned homogeneous, isotropic and linear elastic material properties. The fibre-reinforced matrix structure of the PDL was represented in these models in a similar method to that used in an earlier model [42]. Although the models contain three teeth, fibres were added only to the PDL around the first molar since this is the tooth on which the loads were applied. The PDL fibres were represented by tension-only three-dimensional spar elements (LINK10) [56]. These link elements connected nodes on the interface between the alveolar bone and the PDL with corresponding nodes on the interface between the tooth root and PDL. Each link element was crossed by another link element to represent the criss-cross structure of the PDL fibres [14, 64]. The orientation of the link elements in different regions of the PDL were chosen to represent crestal, horizontal, oblique and apical PDL fibres. In total, 2,112 link elements were added (Fig 3).

The first model to be developed was the most complex fibrous PDL and trabecular structure model. The material properties for the other models were then determined by optimisation so that the tooth displacement for each model matched the displacement in the fibrous PDL trabecular structure model. Therefore, the effective elastic modulus of the solid PDL was the same as the fibrous PDL, and the effective elastic modulus of the trabecular structure model was the same as the bulk trabecular material model. From this it follows that any differences observed in the strain values between models could then be attributed to structural differences (e.g. the presence or absence of PDL fibres, or the presence or absence of trabeculae), rather than to material property differences. The material properties assigned to each material in the models are shown in Table 1 (see S1 Appendix for details of the optimisation process and how material properties were assigned).

**Table 1:** Mechanical properties assigned to each material in all six different models.

Material		Finite Element Model					
		Fibrous PDL, Trabecular Structure	Fibrous PDL, Bulk Material	Solid PDL, Trabecular Structure	Solid PDL, Bulk Material	No PDL, Trabecular Structure	No PDL, Bulk Material
**Cortical Bone**	**Young’s Modulus (MPa)**	17,000	17,000	17,000	17,000	17,000	17,000
	**Poisson’s Ratio**	0.3	0.3	0.3	0.3	0.3	0.3
**Trabecular Bone**	**Young’s Modulus (MPa)**	17,000	526	17,000	526	17,000	526
	**Poisson’s Ratio**	0.3	0.3	0.3	0.3	0.3	0.3
**Trabecular Filling**	**Young’s Modulus (MPa)**	1 x 10^−4^	526	1 x 10^−4^	526	1 x 10^−4^	526
	**Poisson’s Ratio**	0.3	0.3	0.3	0.3	0.3	0.3
**Teeth**	**Young’s Modulus (MPa)**	17,000	17,000	17,000	17,000	17,000	17,000
	**Poisson’s Ratio**	0.3	0.3	0.3	0.3	0.3	0.3
**PDL Matrix**	**Young’s Modulus (MPa)**	1	1	49	49	17,000	17,000
	**Poisson’s Ratio**	0.45	0.45	0.45	0.45	0.3	0.3
**PDL Fibres**	**Young’s Modulus (MPa)**	1,000	1,000	N/A	N/A	N/A	N/A
	**Poisson’s Ratio**	0.35	0.35	N/A	N/A	N/A	N/A

### 2.2 Occlusal load

To provide a straightforward visual assessment of the results from the different models, pair-wise comparisons were made by subtracting the element strains in one model from those in a second model, and presenting the results as colour-coded difference plots. This was done for both maximum (tensile) and minimum (compressive) principal strains.

To provide a more precise comparison of the results, maximum and minimum nodal principal strains were also plotted and compared for the different models. Strain magnitudes were extracted from nodes on the outer surface of the cortical bone along a plane in the buccolingual direction through the centre of where the 500N load was applied. Extracted strains were plotted from nodes on the buccal and lingual sides of the mandibular corpus, from the top down to the lowest node on the inferior surface. The fact that we obtained strains from the outer surface of the cortical bone for the occlusal loads allowed us to study if the ways in which the PDL and trabecular tissue are modelled only affects strains locally, *i*.*e*. in the bones surrounding the PDL, or whether it also affects bone further away from the PDL.

### 2.3 Orthodontic loads

To compare the results from the two orthodontic loads, nodal strain values were selected from the inside surface of the alveolar bone, *i*.*e*. adjacent to the PDL, around both the mesial and distal roots of the loaded molar. For the mesiodistal load, results were obtained from nodes on the mesial and distal sides of each tooth root. Similarly, for the buccolingual load, results were obtained from nodes on the buccal and lingual sides of each tooth root. In both cases, the nodes (on the surface of the alveolar bone) were located on a plane approximately through the apex of the tooth roots. Again, maximum and minimum principal strains were extracted from each node and were plotted against those from other models.

## 3. Results

### 3.1 Occlusal load

#### 3.1.1 Trabecular tissue modelling

**Fig 4:**
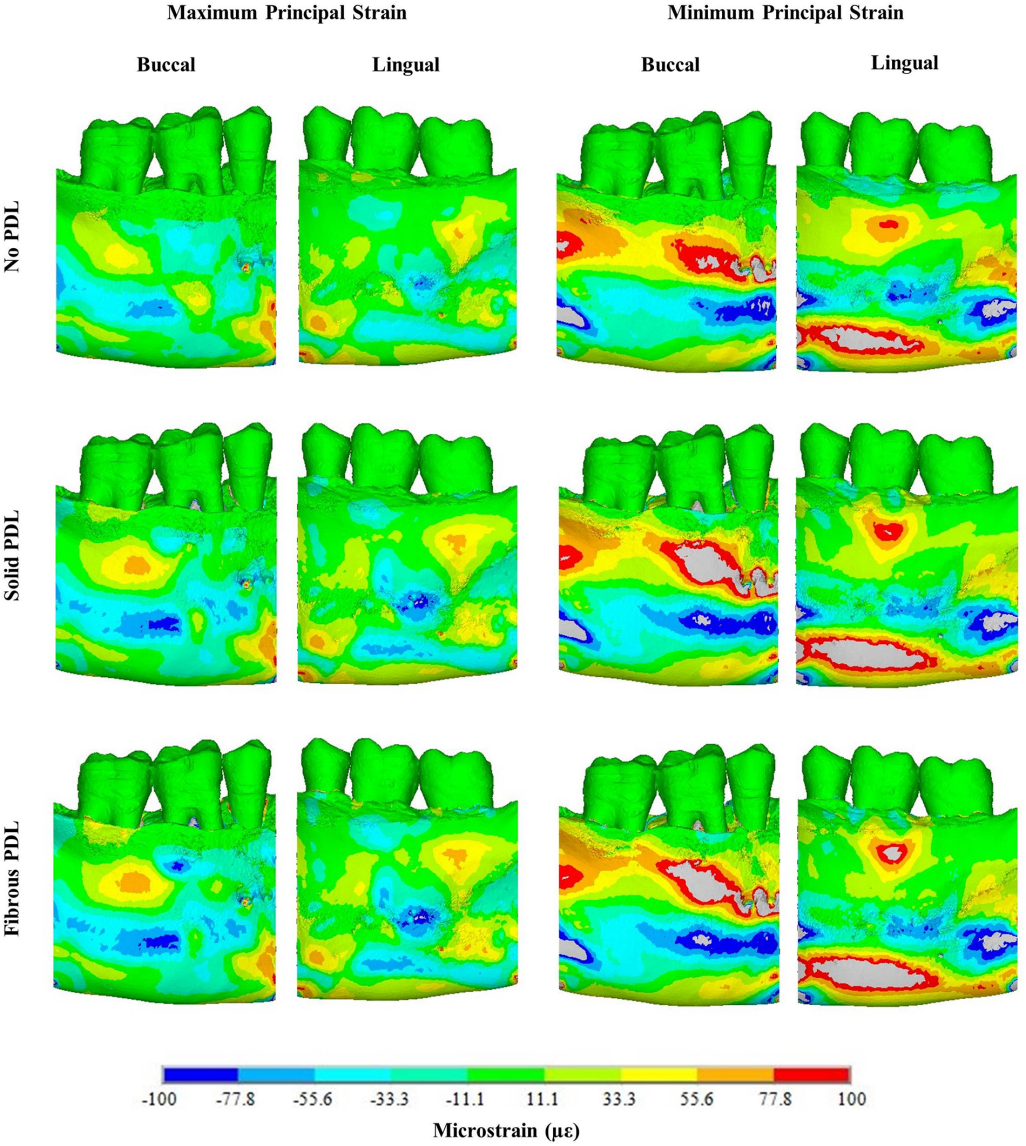
Maximum and minimum principal strain difference plots for the strain differences (in microstrain) between the trabecular structure and bulk trabecular material models with each of the three PDL types. Note, in each case strain from the trabecular structure model is subtracted from strain in the bulk trabecular material model. Therefore, for maximum principal strain negative values indicate strain is higher in the trabecular structure model, whereas for minimum principal strain positive values indicate strain is higher in the trabecular structure model.

**Fig 5:**
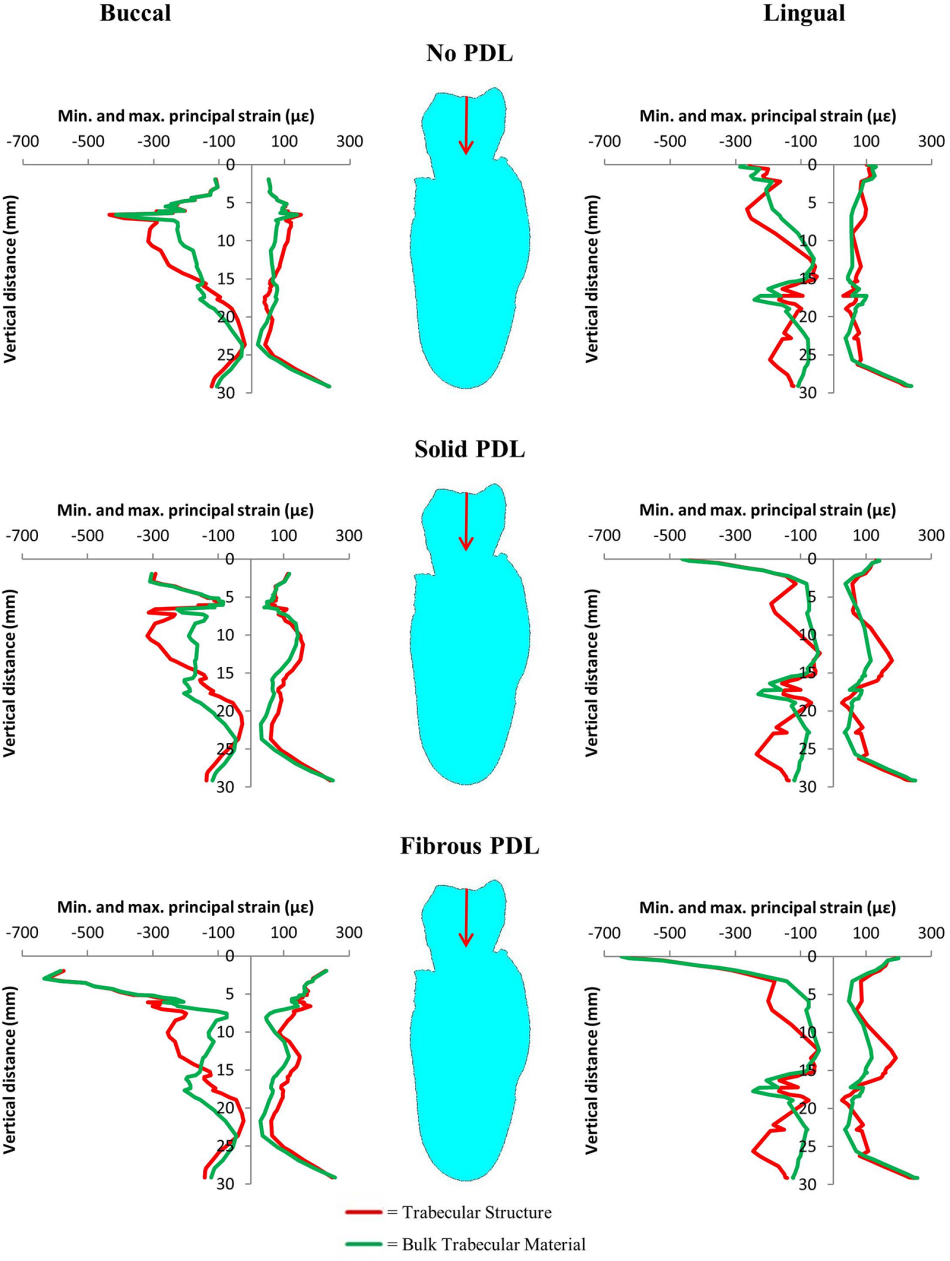
Vertical strain profiles for maximum and minimum principal strains on the buccal and lingual surfaces of the cortical bone from the 500 N occlusal load. Graphs compare results from trabecular structure and bulk trabecular material models for each of the three PDL representations. The red arrows indicate the direction of the applied load.

Difference contour plots comparing maximum principal strains and minimum principal strains for bulk trabecular material and trabecular structure models are shown in Fig 4. For all difference plots, the strain from the trabecular structure model is subtracted from the strain in the bulk trabecular material model. Therefore, for maximum principal strain plots, negative values (cold colours) mean the tensile strain is greater in the trabecular structure model and positive values (warm colours) mean the tensile strain is greater in the bulk trabecular material model. Conversely, for minimum principal strain plots, negative values mean the compressive strain is greater in the bulk trabecular material model and positive values mean the compressive strain is greater in the trabecular structure model. Fig 5 compares the actual nodal strain values on the buccal and lingual surfaces of the cortical bone for the trabecular structure and bulk trabecular material models for each of the three PDL types.

The difference plots for all three PDL types (Fig 4) show that the way in which the trabecular tissue is modelled affects the strain throughout the modelled region of the mandibular corpus and is not confined to a particular area. Tensile strain is greater in the trabecular structure model although there are regions where it is greater in the bulk trabecular material model (Figs 4 and 5). The differences in compressive strain are more pronounced, in some regions strain magnitudes differ between the trabecular models by up to 100% (Figs 4 and 5). Compressive strain is generally higher in the trabecular structure model around the alveolar process and around the inferior portion of the mandible, whereas it is generally higher in the bulk trabecular material model around the mandibular body.

#### 3.1.2 PDL modelling

**Fig 6:**
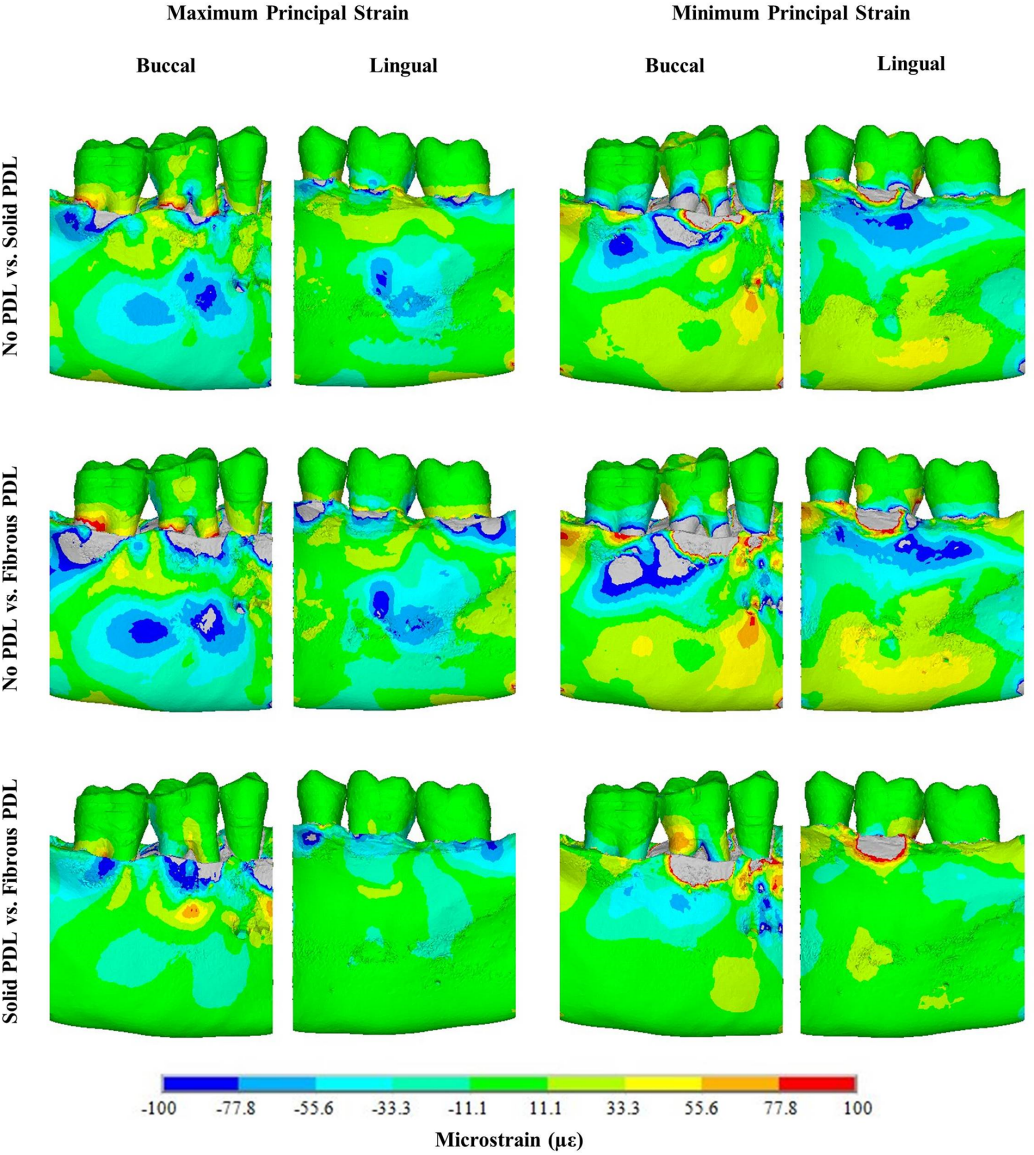
Maximum and minimum principal strain difference plots for the strain differences (in microstrain) between models with trabecular structure trabecular tissue, but different PDLs as indicated. Note, in each case strain in the second model is subtracted from strain in the first model. Therefore, for maximum principal strain negative values indicate strain is higher in the second model, whereas for minimum principal strain positive values indicate strain is higher in the second model.

**Fig 7:**
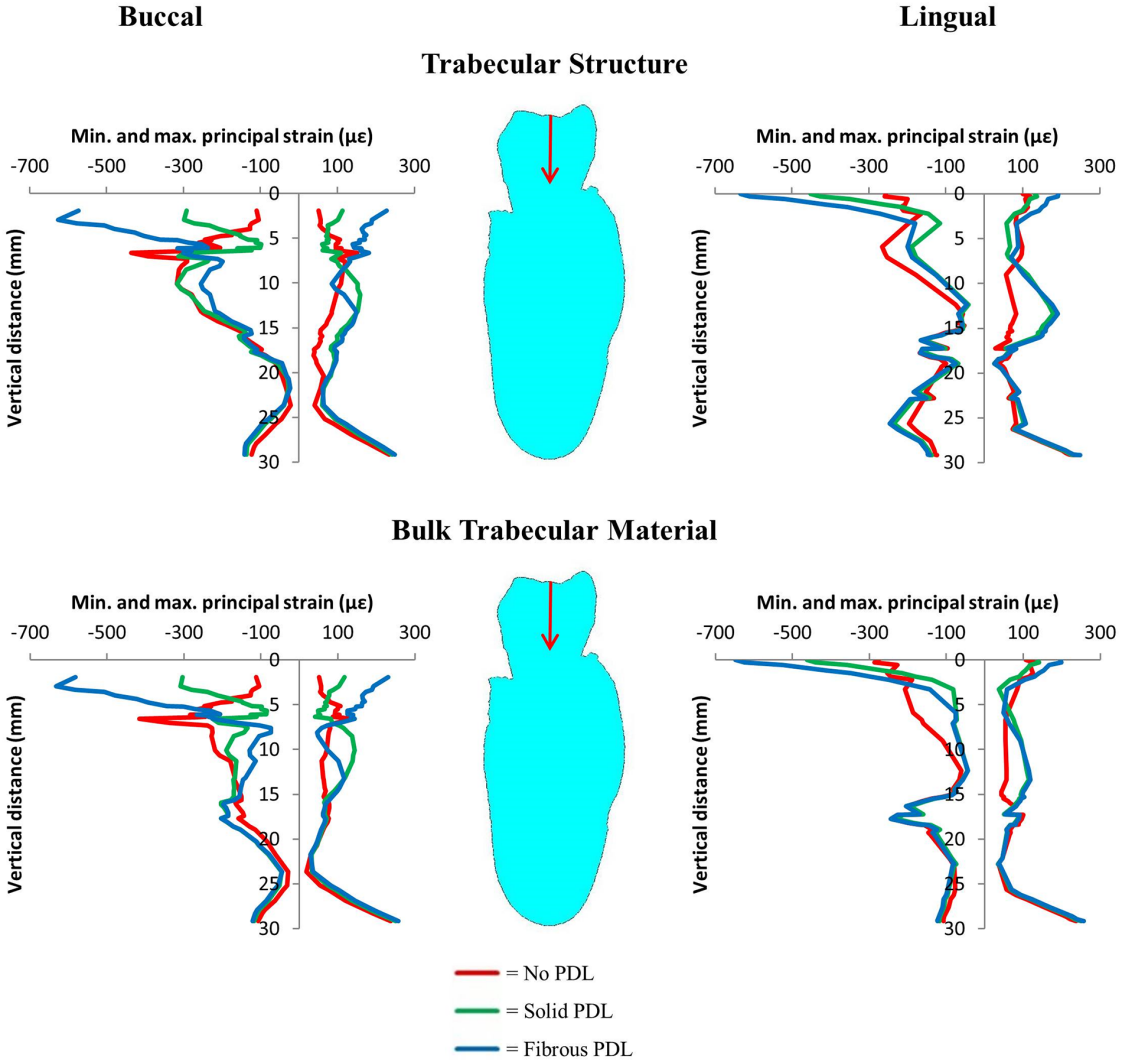
Vertical strain profiles for maximum and minimum principal strains on the buccal and lingual surfaces of the cortical bone from the 500 N occlusal load. Graphs compare results from no PDL, solid PDL and fibrous PDL models for both trabecular tissue types. The red arrows indicate the direction of the applied load.

Fig 6 shows difference contour plots comparing principal strain magnitudes between the three PDL types in the trabecular structure models (see S2 Appendix for the difference plots from the bulk trabecular material models). Although there are three types of PDL, only two models can be compared at a time in a difference contour plot, *e*.*g*. no PDL vs solid PDL. In each case, strains in the second model have been subtracted from strains in the first model. As in Fig 4, negative values (cold colours) in the maximum principal strain plots mean the tensile strain is greater in the second model, whereas for minimum principal strain difference plots, negative values mean compressive strain is greater in the first model. Fig 7 compares nodal strain results from each of the three PDL types for both trabecular tissue types.

The results in Fig 6 (trabecular structure models) are similar to those in S2 Appendix (bulk trabecular material models). In each of these figures, the difference plots comparing the no PDL and solid PDL models are similar to those comparing the no PDL and fibrous PDL models. Tensile strain is higher in models with a PDL (solid and fibrous) than in the model without PDL. There is more variation in the results for compressive strain, with some regions of the model showing higher strain with a PDL and some showing higher strain with no PDL. This is also shown by the strain graphs in Fig 7, where, other than for compressive strain around the alveolar process, strain is lower in the no PDL model than in the two models with PDL. In the superior part of the mandibular corpus, both compressive and tensile strain, though especially compressive strain, are 100–500% higher in models with a PDL than without, and higher in the fibrous PDL model than in the solid PDL model.

Comparing the solid PDL and fibrous PDL models, there are large differences in the alveolar process with the fibrous PDL model generally having the higher strains. However, for most of the mandibular body, especially on the lingual side, the strains are similar. In general, the plots show that strain magnitudes differ more between the no PDL model and either of the two models with a PDL (either solid or fibrous), than between the solid PDL and fibrous PDL models.

### 3.2 Orthodontic loads

**Fig 8:**
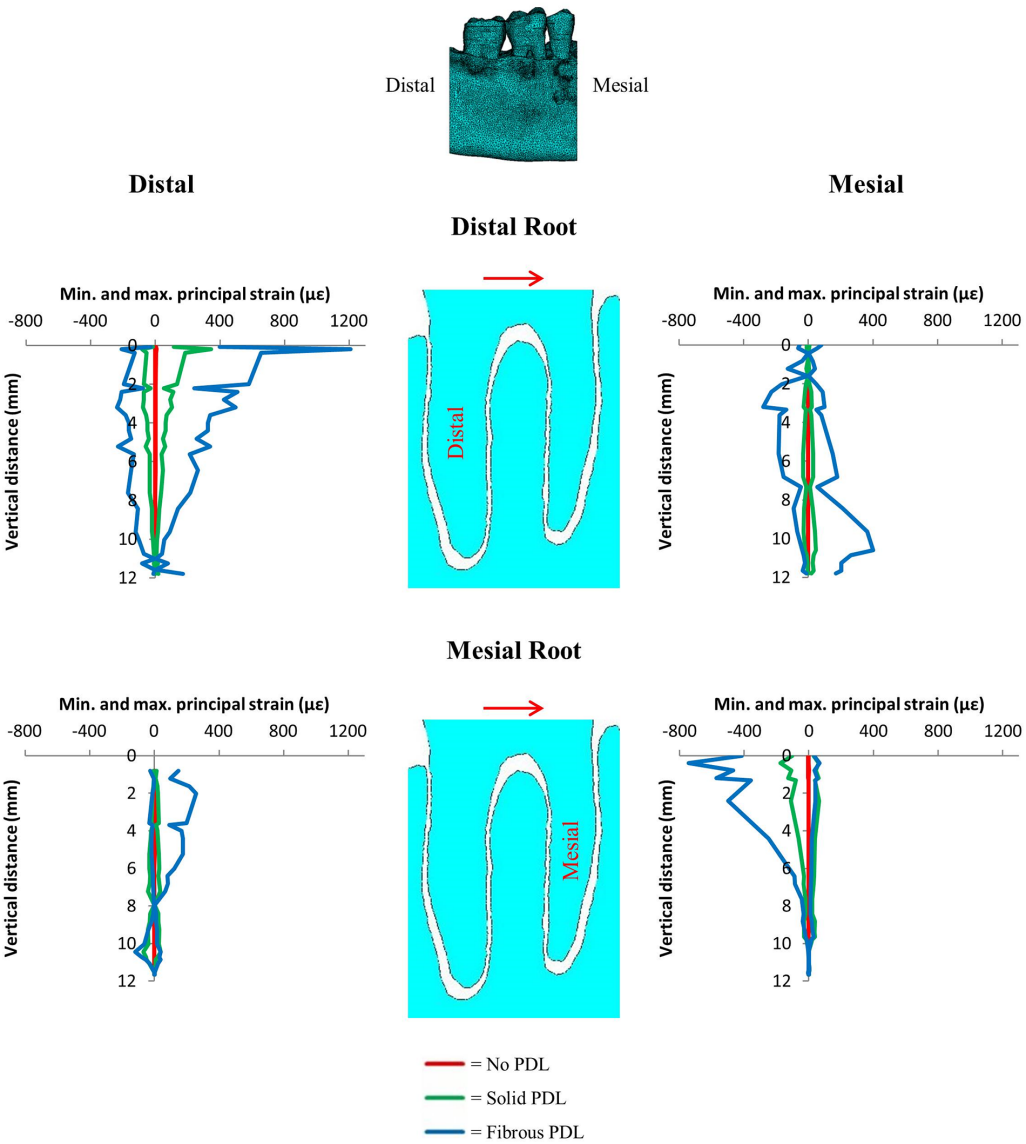
Vertical strain profiles for maximum and minimum principal strains on the distal and mesial surfaces of the alveolar bone around both the distal and mesial tooth roots from the 1 N distomesial orthodontic load. Graphs compare results from no PDL, solid PDL and fibrous PDL models each with trabecular structure trabecular tissue. The red arrows indicate the direction of the applied load. The image at the top indicates the viewing direction for the results where the distal and mesial sides of the model are identified.

**Fig 9:**
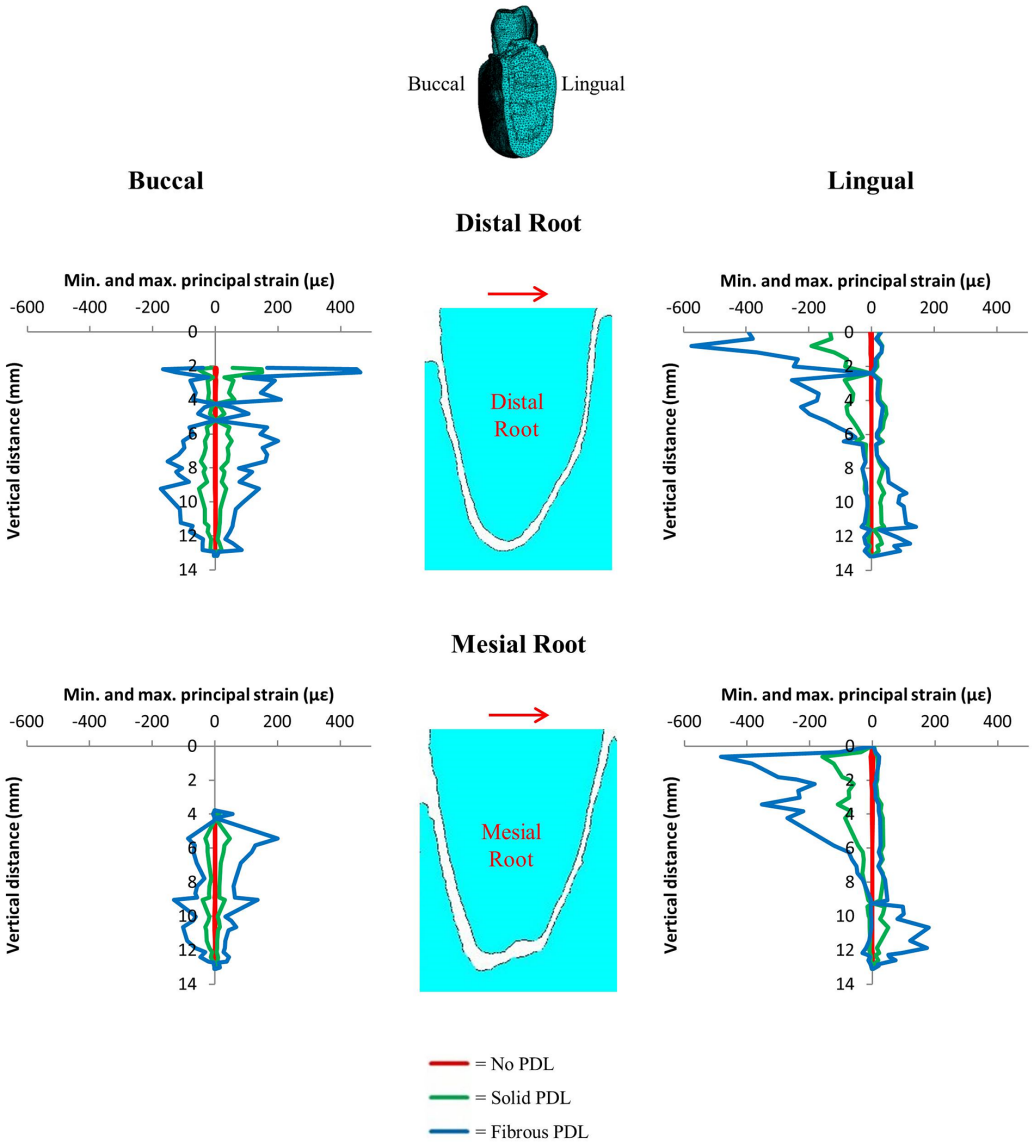
Vertical strain profiles for maximum and minimum principal strains on the buccal and lingual surfaces of the alveolar bone around both the distal and mesial tooth roots from the 1 N buccolingual orthodontic load. Graphs compare results from no PDL, solid PDL and fibrous PDL models each with trabecular structure trabecular tissue. The red arrows indicate the direction of the applied load. The image at the top indicates the viewing direction for the results where the buccal and lingual sides of the model are identified.

Figs 8 and 9 show the nodal strain values for each of the three PDL types in the trabecular structure model for the mesiodistal and the buccolingual orthodontic load respectively (see S3 and S4 Appendices for the very similar strain values obtained from the bulk trabecular material model). The strain magnitudes for each of the PDL representations are significantly different with values between 0 and ±10*με* for the no PDL model, but strains of one to two magnitudes higher for the models including PDL (solid and fibrous). The fibrous PDL model shows the highest strain magnitudes of the three models. However, despite the large differences in strain magnitudes, there are some similarities in the spatial distribution of high versus low strain magnitudes between the fibrous and the solid PDL model (S3 and S4 Appendices).

## 4. Discussion

To the best of our knowledge, this is the first FEA study to include PDL fibres together with realistic μCT-based tooth and bone geometry. The results from this study show that modelling the fibrous structure of the PDL leads to different strains in the alveolar bone compared to modelling it as a layer of solid material. This is particularly the case when orthodontic loads are simulated. Strains in the bone are increased when considering the fibrous structure of the PDL. In addition, the results support the findings of previous studies such as [9] that failing to include the PDL in mandibular FE models reduces the strains not only in the alveolar bone but also the cortical bone further away from the PDL.

### 4.1 Trabecular tissue modelling

The similarities between the difference plots for the three PDL representations (Fig 4) suggest that the way in which the trabecular tissue is modelled affects the strain magnitudes in the bone irrespective of how the PDL is modelled, and that strain magnitudes increase when the trabecular structure is modelled instead of using a bulk trabecular tissue model. Previous studies have suggested that it is the cortical bone rather than the trabecular bone which is primarily responsible for resisting masticatory loads in the mandible [65, 66]. This is unsurprising when the smaller volume of the trabecular bone resulting from the porosity of the trabecular tissue is considered. However, the presence of trabeculae rather than a bulk filler will alter the load transfer path between the tooth and cortical bone. This altered load path is a likely explanation for the differences in strain between the trabecular structure and the bulk trabecular material model that we observed.

In contrast, the comparisons of the trabecular structure and bulk material models for the orthodontic loads (S3 and S4 Appendices) showed very little difference between the two types of trabecular tissue models. This finding is likely to be explained by the direction of the orthodontic loads since they are orientated in the transverse plane, so that the load does not pass directly through the trabecular bone as it does with the occlusal load.

### 4.2 PDL modelling—Occlusal load

For the different types of PDL, the results from the difference plots (Fig 6 and S2 Appendix) and the strain graphs (Fig 7) show that strains are more similar in the two models with PDL (solid or fibrous) than in the no PDL model. This suggests that it is more important to include a PDL layer in mandibular FE models than to model its fibrous structure. Other than for compressive strain around the alveolar process, strain is generally higher in the models with a PDL. This agrees with the findings of Gröning *et al*. [18] and Marinescu *et al*. [2] which both found that not including the PDL increases the stiffness of the mandible and thus decreases the strains observed. It should be noted again that the strains observed here (Figs 5 and 7) are not typical strains expected during daily occlusal loading, but rather at the higher end of the spectrum since a maximum bite force was used. However, it is the relative differences between the models and not the absolute strain values which are of interest here.

Although there are differences in strains observed with and without a PDL, the strain distribution is generally quite similar for all three types of PDL, especially towards the base of the mandible furthest away from the PDL (Fig 7). This may be due to the boundary conditions applied. The loading applied to the section of mandible is equivalent to a three-point bending situation, with the two ends supported and a load applied in the middle. Therefore, the resultant bending will put the mandible in tension at the bottom and compression at the top, as shown by the strains in Fig 7. Beam mechanics suggests that in this situation, the effect of including the PDL would be less than if the direction of bending was reversed [18]. Gröning *et al*. [18] also found that including the PDL in human mandibular FE models led to increased torsion of the mandible about the posterior-anterior axis compared to models without a PDL. In addition, Hylander [67, 68] found that, in non-human primates, wishboning was the main type of deformation observed in the mandible during mastication. Since only a section of the molar region was modelled in our study, rather than the whole mandible as *e*.*g*. in Gröning *et al*. [18], we did not include loading regimes such as torsion or wishboning, so that we are likely to underestimate the effects of including PDL on the strains in the mandibular corpus.

### 4.3 PDL modelling—Orthodontic load

The results show that there is a large difference in strain magnitude predicted for each of the three different types of PDL for both orthodontic loads (Figs 8 and 9). This is especially true for the no PDL model which has strains typically two to three orders of magnitude lower than that of the fibrous PDL model. This is because without PDL the tooth is fused with the alveolar bone creating a rigid structure. There is less difference between the solid PDL and fibrous PDL models, however, the strains in the fibrous PDL model are still typically two to three times greater than the strains in the solid PDL model.

The difference plots and strain graphs showed that the solid and fibrous PDLs produced similar results (Figs 6 and 7). However, with the orthodontic loads this was not the case (Figs 8 and 9). Young’s modulus for the solid PDL was optimised to match that of the fibrous PDL under the occlusal load since that would be the dominant load which occurs naturally, whereas orthodontic loads are not natural. Furthermore, the PDL fibres are arranged primarily to provide axial and torsional stability rather than stability in the buccolingual or mesiodistal directions. Shear forces resulting from transverse movements during chewing would cause some natural tooth movement in those directions, however, this would be combined with an occlusal load which would stiffen the PDL fibre system. So, the two models are similar under the physiological occlusal load, whereas the fibrous PDL provides less resistance in the case of the non-physiological orthodontic load.

During orthodontic tooth movement, the tooth moves in the direction of the applied load, *i*.*e*. from left to right in both Figs 8 and 9. From these models, considering the distribution of compressive versus tensile strains (Figs 8 and 9), for orthodontic tooth movement to occur, bone resorption would have to occur in areas under compression and bone formation in areas under tension. Therefore, our results most closely agree with the pressure-tension hypothesis [38]. However, this is not how bone adaptation is generally thought to occur: it is usually thought that compression causes bone formation and tension causes bone resorption [69, 70].

To overcome this apparent contradiction, it is suggested that orthodontic tooth movement is controlled by the PDL rather than by alveolar bone, and this has been supported by biological studies examining cellular activity within the PDL [37, 71]. An additional reason for suggesting that orthodontic tooth movement is controlled by the PDL, rather than the alveolar bone, is that strain in the alveolar bone is thought to be far below that which would typically be required for mechanical adaptation of bone to occur [36, 43, 45, 46]. However, based on synchrotron scans of alveolar bone samples which showed the surface of the alveolar bone to be rough, Dalstra et al. [47, 48] suggested that previous FEA studies may have grossly underestimated the strain in the alveolar bone by modelling smooth PDLs and tooth sockets. The models presented here include more accurate representations of the tooth sockets, including the rough bone surface, and do show some areas of high strain (Figs 8 and 9). However, these high strains only occurred in limited regions, and rapidly decreased when moving towards the root apex.

With regards to orthodontic tooth movement, there were several limitations in this study. Firstly, only bone strains were considered when discussing how the different PDL types affect load transfer to the tooth socket. Since strains in the PDL have also been suggested as a trigger for orthodontic tooth movement [37], future research could consider a more detailed examination of strain in the PDL, especially with fibrous PDLs. Additionally, only linear elastic material properties were considered. This is a common simplification in dental FE models [21], despite the fact the PDL is known to be viscoelastic [72]. Previous authors have included the nonlinear material properties of the PDL [17, 22, 23], but viscoelasticity was not considered here since we could not be confident on the relative properties of the fibre and bulk material or loading rate, and using potentially arbitrary values could confound the primary purpose of the study to identify the effect of including the PDL fibres. Finally, orthodontic tooth movement is an iterative process, but only initial strains are plotted here. Adaptive remodelling algorithms have been used to simulate orthodontic tooth movement [46], so future work could also consider the inclusion of fibrous PDLs in models which simulate orthodontic tooth movement.

## 5. Conclusions

The primary aim of this study was to investigate the effect of including the PDL in mandibular FE models, to determine how PDL affects the load transfer between the tooth and mandibular bone and whether it is necessary to include details of its fibrous structure, rather than representing PDL as a simple solid layer. For the first time, we have modelled PDL fibres together with realistic, rather than simplified, tooth and bone geometry. In addition, two different methods of modelling the trabecular tissue were investigated.

Including PDL in FE models adds significantly to the time required to both create and solve the model. The results here indicate that it is important to include the PDL in mandibular FE models when either occlusal or orthodontic loads are considered. Failure to include the PDL fuses the teeth to the alveolar bone creating a much more rigid structure, leading to reduced strains throughout the model not just in the area around the PDL. This agrees with the findings from previous studies, for example [2, 9, 18].

Modelling the fibrous structure of the PDL increased strains in the alveolar bone considerably compared to a solid PDL when orthodontic loads were simulated. This may be important when considering the biomechanical stimuli for alveolar bone remodelling as previous finite element studies have found the strains in the alveolar bone to be very low [36, 43–45] and thus might have underestimated the strains in the alveolar bone [47, 48]. Therefore, future FEA models that include the fibrous structure of the PDL as well as an accurate representation of bone morphology could be very useful in helping to improve our understanding of orthodontic tooth movement.

## Supporting information

S1 Appendix Material properties and optimisation. (DOCX)

S2 Appendix Additional occlusal load results. (DOCX)

S3 Appendix Additional mesiodistal orthodontic results. (DOCX)

S4 Appendix Additional buccolingual orthodontic load results. (DOCX)
