# Supplementary material for: Inclusion of periodontal ligament fibres in mandibular finite element models leads to an increase in alveolar bone strains
Source: PLoS One. 2017 Nov 30;12(11):e0188707. doi: 10.1371/journal.pone.0188707 (PMC5708643; doi:10.1371/journal.pone.0188707)
Supplement: S1 Appendix — (DOCX) [file pone.0188707.s001.DOCX]

**Appendix 1**

**Material Properties and Optimisation**

Having defined the boundary conditions, the six different models were created by varying the material properties assigned to the different elements, with or without the addition of PDL fibres. The first model to be developed was the most anatomically accurate fibrous PDL and trabecular structure model. The material properties for the other models were then determined by optimisation, so the tooth displacement for each model was matched to that of the fibrous PDL trabecular structure model.

The material properties of cortical bone and of the bone forming the individual trabeculae are generally thought to be similar (Currey, 1989, 2002). Therefore, in models where the trabecular tissue was modelled as a detailed trabecular structure, the trabecular bone and cortical bone were given the same material properties, with the trabecular filling being given a very low Young’s modulus value. In models where the trabecular tissue was then modelled as a bulk material, both the trabecular bone and trabecular filling were given the same material properties, but with a Young’s modulus value much lower than that of cortical bone.

Cortical bone, trabecular bone and, for simplicity, teeth were all assigned a Young’s modulus of 17,000 MPa with a Poisson’s ratio of 0.3 (Gröning *et al.*, 2011a). The teeth were not separated into different components but were modelled entirely as dentin, which has similar properties to bone (Currey, 2002). It was not necessary to include enamel since the strains within the teeth are not of interest in this study. The purpose of including the trabecular filling was to allow the same finite element mesh to be used for both the trabecular structure and bulk material models. To ensure the filling had a negligible effect in the trabecular structure model, the filling was given a very low Young’s modulus of 1 x 10^-4^ MPa; (this was confirmed in a separate sensitivity study, not reported here).

For the fibrous PDL models, the material properties of the PDL matrix and PDL fibres were chosen to be the same as those used previously (McCormack *et al.*, 2014) which were obtained from previous studies. Thus, the PDL matrix was given a Young’s modulus value of 1 MPa with a Poisson’s ratio of 0.45, and the PDL fibres were given a Young’s modulus value of 1,000 MPa, with a Poisson’s ratio of 0.35. For the PDL fibres, it was also necessary to specify the cross-sectional area and (optional) initial strain of the link elements. Since approximately 50 to 75% of the PDL tissue volume consists of the PDL fibres (Dorow *et al.*, 2003), the cross-sectional area of the link elements was calculated so that their combined volume was within this value. As a result, the cross-sectional area was chosen to be 0.06 mm^2^. This is one to two orders of magnitude thicker than the collagen fibres found *in vivo* (Berkovitz, 1990; Meyer *et al.*, 2010), but of course the number of modelled PDL fibres was significantly less than the number found *in vivo*. As no data were available in the literature to suggest a suitable value for any initial strain of the fibre elements, this value was defined as zero.

Thus, in summary, the material properties of the fibrous PDL trabecular structure model are presented in Table 1. The remaining models were then produced by altering the material properties assigned to different materials and optimising the necessary Young’s modulus value in order to match the tooth displacement of the new model to that of the core fibrous PDL trabecular structure model. For this, tooth displacement was defined as the average vertical displacement of the nodes to which the occlusal load had been applied. For the occlusal force of 500N, this was found to be 0.0593 mm. This value is similar to the tooth displacement calculated for a simplified single tooth model (0.0703 mm in McCormack *et al.* (2014)), and seems reasonable when compared to the value of 0.12mm cited by Borák *et al.* (2011) from an experiment by Kato (1982).

**Table 1 Mechanical properties assigned to each material in the original Fibrous PDL Trabecular Structure model.**

| **Component** | **Young’s Modulus (MPa)** | **Poisson’s Ratio** |
| --- | --- | --- |
| Cortical bone^a^ | 17,000 | 0.30 |
| Trabecular bone^b^ | 17,000 | 0.30 |
| Trabecular filling | 1 x 10^-4^ | 0.30 |
| Teeth^a^ | 17,000 | 0.30 |
| PDL matrix^c^ | 1 | 0.45 |
| PDL fibres^d^ | 1,000 | 0.35 |

^a^ Gröning *et al.* (2011).

^b^ Currey (1989, 2002).

^c^ Jones *et al.* (2001), Qian *et al.* (2001).

^d^ Gautieri *et al.* (2012), Katona and Qian (2001), Meyer *et al.* (2010), Rees and Jacobsen (1997).

The purpose of this study was to investigate three different ways of modelling the PDL (fibrous PDL, solid PDL and no PDL), and two different ways of modelling the trabecular tissue (trabecular structure and bulk trabecular material). Each of the PDL types were modelled with both of the two trabecular tissue types meaning there were a total of six different models created. So, the only difference between the trabecular structure model and the bulk trabecular material model, with the same PDL, was the way in which the trabecular tissue was modelled. Similarly, the only difference between fibrous PDL, solid PDL and no PDL models, with the same trabecular tissue, was the way in which the PDL was modelled. The tooth displacement for the two fibrous PDL models and the two solid PDL models were therefore matched by a series of optimisations. Since the majority of tooth displacement is due to deformation of the PDL (Naveh *et al.*, 2012), tooth displacement was not matched to the original for the no PDL models.

The process was as follows (see Figure 1). The first optimisation was used to create the fibrous PDL bulk trabecular material model. For this, the trabecular bone and trabecular filling material were assigned the same material property and their common Young’s modulus value varied until the tooth displacement for the new model matched that of the original model. To achieve the same displacement, it was found necessary to use a Young’s modulus value of 526 MPa for the two materials. This value is within the range of reported values for Young’s modulus of bulk trabecular bone (Van Eijden, 2000) and is similar to values used in other finite element models (e.g. Gröning *et al.*, 2012).

The second optimisation was to create the solid PDL trabecular structure model. This model differed from the original model only in how the PDL was modelled. For this model, the link elements representing the PDL fibres were removed from the model leaving only a solid material to represent the PDL. The Young’s modulus value assigned to the PDL was varied until the tooth displacement for this model matched that of the original model. To get the same displacement, it was found necessary to use a Young’s modulus value of 49 MPa. Again this value is within the range of reported values for Young’s modulus of PDL (Rees, 2001; Fill *et al.*, 2011) and is similar to values used in other finite element models (*e.g.* Rees & Jacobsen, 1997).

The third optimisation was to create the solid PDL bulk trabecular material model. In the second optimisation, the effective elastic modulus of the solid PDL was matched to that of the fibrous PDL, so this value was used again for this model. The trabecular bone and trabecular filling were then assigned the same common material property and their Young’s modulus value was varied until the tooth displacement again matched that of the original solid PDL trabecular structure model. To get the same displacement, it was again found necessary to use a Young’s modulus value of 526 MPa for the two materials. This was the same value found in the first optimisation, and thus the overall effective elastic modulus of the trabecular tissue was the same for these two models.

Fibrous PDL

trabecular structure

Solid PDL

trabecular structure

**E_PDL_ = 49 MPa**

Solid PDL

bulk trabecular material

**E_TrabecularTissue_ = 526 MPa**

Fibrous PDL

bulk trabecular material

**E_TrabecularTissue_ = 526 MPa**

**START**

Optimisation 1

Optimisation 2

Optimisation 3

**Figure 1 Schematic diagram summarising the steps involved in the optimisation process to calculate Young’s modulus values to ensure the same tooth displacement in each model.**

The final two models were the no PDL trabecular structure and no PDL bulk trabecular material models. These models were simply created from the corresponding solid PDL models by changing the material properties of the PDL material to be the same as cortical bone. This is the same method as that used to create the no PDL models in McCormack *et al.* (2014), and is the same method adopted by Gröning *et al.* (2011) to compare no PDL and solid PDL models.

**References**

Berkovitz, B. K. B. (1990). The structure of the periodontal ligament: an update. *European Journal of Orthodontics* 12, 51-76.

Borák, L., Florian, Z., Bartáková, S., Prachár, P., Murakami, N., Ona, M., Igarashi, Y. and Wakabayashi, N. (2011). Bilinear elastic property of the periodontal ligament for simulation using a finite element mandible model. *Dental Materials Journal* 30, 4, 448-454.

Currey, J. D. (1989). Strain rate dependence of the mechanical properties of reindeer antler and the cumulative damage model of bone fracture. *J. Biomech.* 22, 469-475.

Currey, J. D. (2002). *Bones: structure and mechanics.* Princeton: Princeton University Press.

Dorow, C., Krstin, N. and Sander, F. G. (2003). Determination of the Mechanical Properties of the Periodontal Ligament in a Uniaxial Tensional Experiment. *J. Orofac. Orthop.* 64, 100-107.

Fill, T. S., Carey, J. P., Toogood, R. W. and Major, P. W. (2011). Experimentally Determined Mechanical Properties of, and Models for, the Periodontal Ligament: Critical Review of Current Literature. *J. Dent. Biomech.* 10, 2011, 1-10.

Gautieri, A., Vesentini, S., Redaelli, A. and Buehler, M. J. (2012). Viscoelastic properties of model segments of collagen molecules. *Matrix Biology* 31, 141-149.

Gröning, F., Fagan, M. J. and O’Higgins, P. (2011). The effects of the periodontal ligament on mandibular stiffness: a study combining finite element analysis and geometric morphometrics. *Journal of Biomechanics* 44, 1304-1312.

Gröning, F., Fagan, M. and O’Higgins, P. (2012). Modeling the Human Mandible Under Masticatory Loads: Which Input Variables are Important? *Anat. Rec.* 295, 853-863.

Jones, M. L., Hickman, J., Middleton, J., Knox, J. and Volp, C. (2001). A Validated Finite Element Method Study of Orthodontic Tooth Movement in the Human Subject. *Journal of Orthodontics* 28, 29-38.

Kato, H. (1982). The function of tooth supporting structures. Part II. The dynamics of molars in function and at rest. *J. Jpn. Prosthodont. Soc.* 26, 133-147.

Katona, T. R. and Qian, H. (2001). A mechanism of noncontinuous supraosseous tooth eruption. *Am. J. Orthod. Dentofacial Orthop.* 120, 263-271.

McCormack, S. W., Witzel, U., Watson, P. J., Fagan, M. J. and Gröning, F. (2014). The Biomechanical Function of Periodontal Ligament Fibres in Orthodontic Tooth Movement. *PLoS ONE* 9, 7, e102387.

Meyer, B. N., Chen, J. and Katona, T. R. (2010). Does the center of resistance depend on the direction of tooth movement? *Am. J. Orthod. Dentofacial Orthop.* 137, 354-361.

Naveh, G. R. S., Chattah, N. L. T., Zaslansky, P., Shahar, R. and Weiner, S. (2012). Tooth-PDL-bone complex: Response to compressive loads encountered during mastication – A review. *Archives of Oral Biology* 57, 1575-1584.

Qian, H., Chen, J. and Katona, T. R. (2001). The influence of PDL principal fibers in a 3-dimensional analysis of orthodontic tooth movement. *Am. J. Orthod. Dentofacial Orthop.* 120, 272-279.

Rees, J. S. (2001). An investigation into the importance of the periodontal ligament and alveolar bone as supporting structures in finite element studies. *Journal of Oral Rehabilitation* 28, 425-432.

Rees, J. S. and Jacobsen, P. H. (1997). Elastic modulus of the periodontal ligament. *Biomaterials* 18, 995-999.

Van Eijden, T. M. G. J. (2000). Biomechanics of the Mandible. *Crit. Rev. Oral. Biol. Med.* 11, 1, 123-136.
