# Supplementary material for: Inclusion of periodontal ligament fibres in mandibular finite element models leads to an increase in alveolar bone strains
Source: PLoS One. 2017 Nov 30;12(11):e0188707. doi: 10.1371/journal.pone.0188707 (PMC5708643; doi:10.1371/journal.pone.0188707)
Supplement: S2 Appendix — (DOCX) [file pone.0188707.s002.docx]

**Appendix 2**

**Additional Occlusal Load Results**


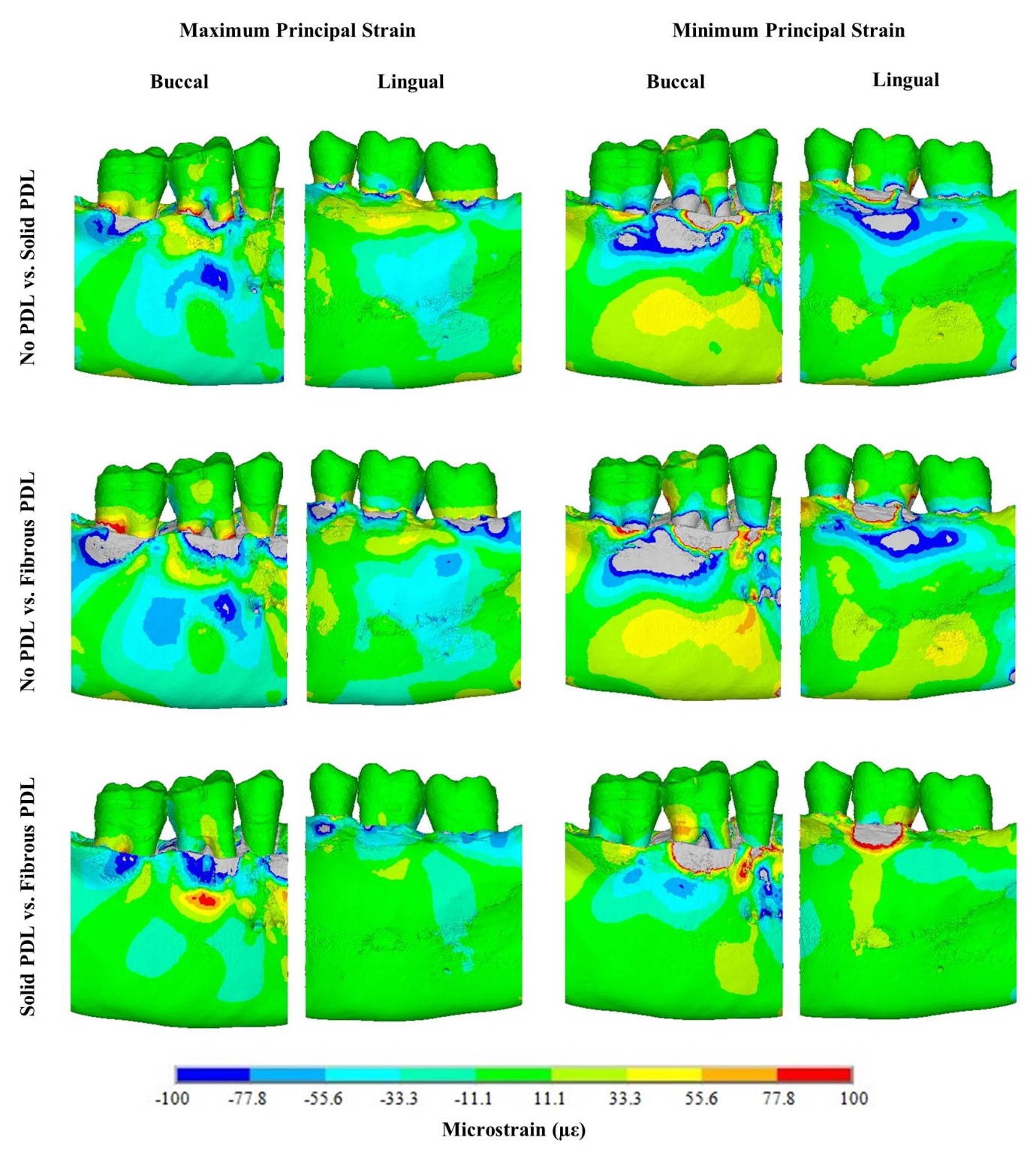


**Fig S2.1. Maximum and minimum principal strain difference plots for the strain differences (in microstrain) between models with bulk trabecular material trabecular tissue, but different PDLs as indicated.** Note, in each case strain in the second model is subtracted from strain in the first model. Therefore, for maximum principal strain negative values indicate strain is higher in the second model, whereas for minimum principal strain positive values indicate strain is higher in the second model.
