# Supplementary material for: Inclusion of periodontal ligament fibres in mandibular finite element models leads to an increase in alveolar bone strains
Source: PLoS One. 2017 Nov 30;12(11):e0188707. doi: 10.1371/journal.pone.0188707 (PMC5708643; doi:10.1371/journal.pone.0188707)
Supplement: S3 Appendix — (DOCX) [file pone.0188707.s003.docx]

**Appendix 3**

**Additional Mesiodistal Orthodontic Load Results**


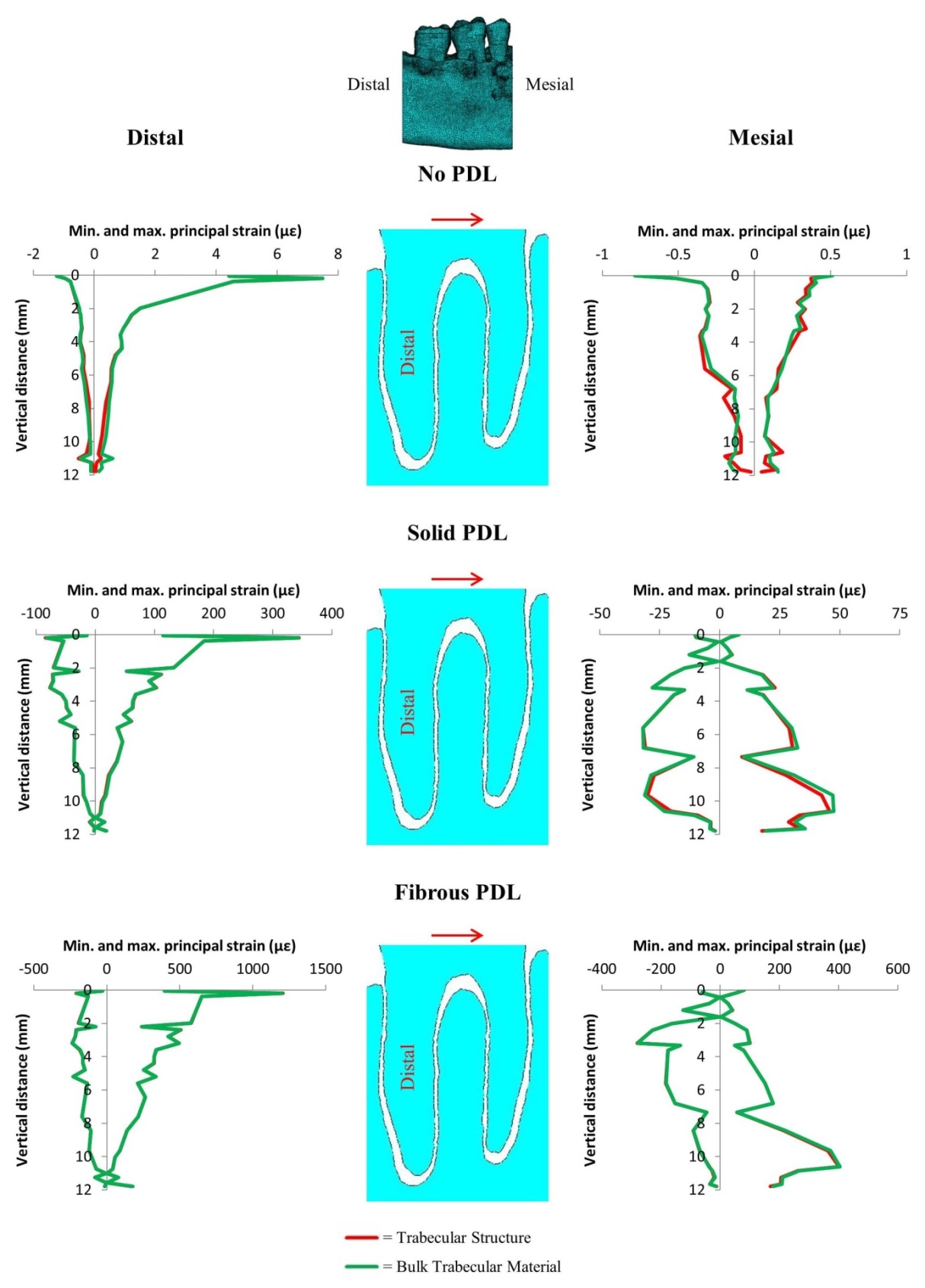


**Fig S3.1. Vertical strain profiles for maximum and minimum principal strains on the distal and mesial surfaces of the alveolar bone around the distal tooth root from the 1 N mesiodistal orthodontic load.** Graphs compare results from trabecular structure and bulk trabecular material models for each of the three PDL types. The red arrows indicate the direction of the applied load, and the image at the top indicates the viewing direction for the results. [Note the different scales of the x-axes].


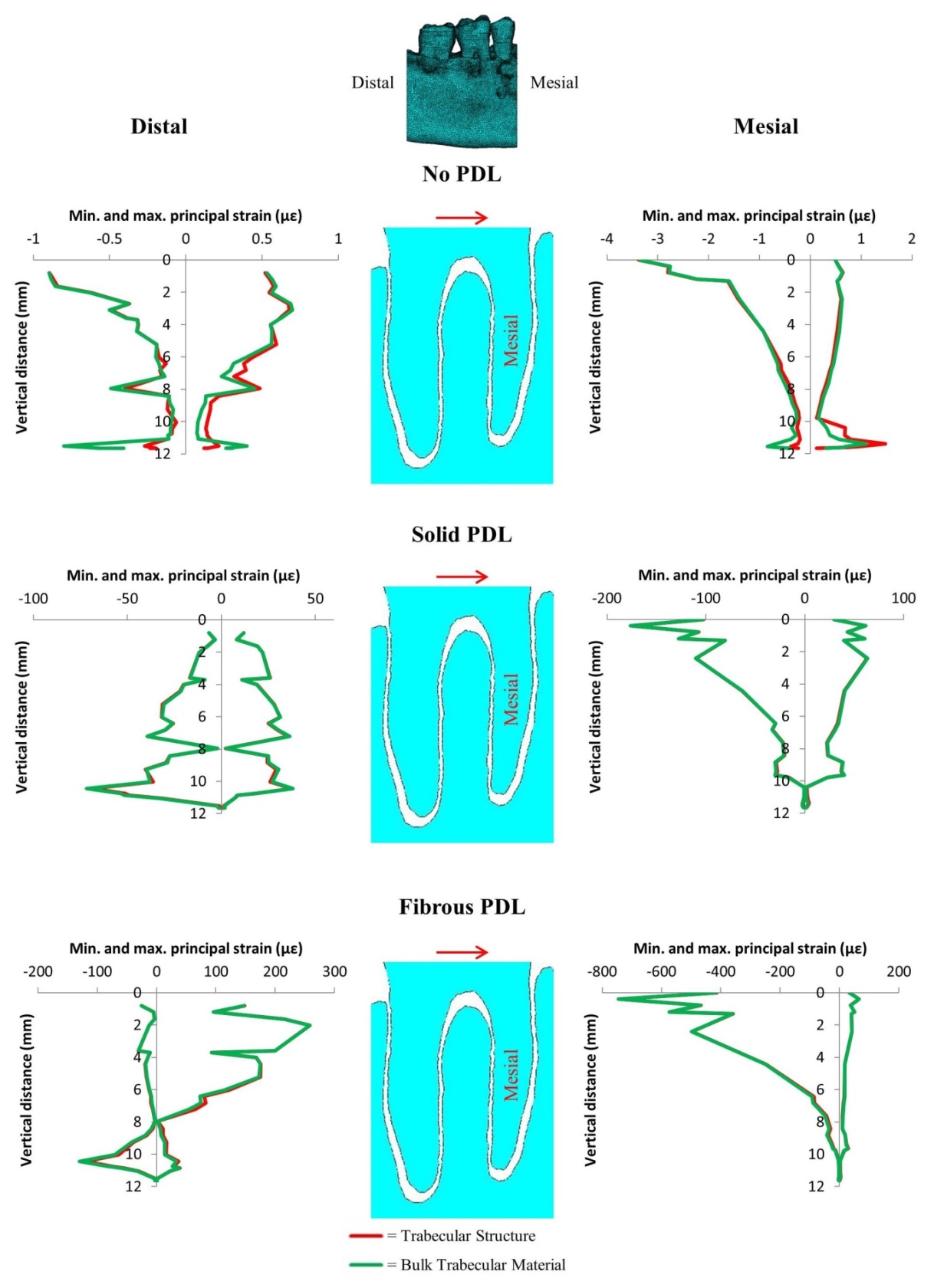


**Fig S3.2. Vertical strain profiles for maximum and minimum principal strains on the distal and mesial surfaces of the alveolar bone around the mesial tooth root from the 1 N mesiodistal orthodontic load.** Graphs compare results from trabecular structure and bulk trabecular material models for each of the three PDL types. The red arrows indicate the direction of the applied load, and the image at the top indicates the viewing direction for the results. [Note the different scales of the x-axes].
